# Autism and Schizotypal Traits in Relation to Thought-Action Fusion in Obsessive-Compulsive Disorder

**DOI:** 10.62641/aep.v54i1.2027

**Published:** 2026-02-15

**Authors:** Vasfiye Ozek, Doga Sevincok, Levent Sevincok

**Affiliations:** ^1^Department of Psychiatry, Aydin State Hospital, 09100 Aydin, Turkey; ^2^Department of Child and Adolescent Psychiatry, Istinye University, 34488 Istanbul, Turkey; ^3^Department of Psychiatry, Aydin Adnan Menderes University, 09100 Aydin, Turkey

**Keywords:** autistic disorder, obsessive-compulsive disorder, schizotypal personality disorder, thought-action fusion

## Abstract

**Background:**

Thought-Action Fusion (TAF) is one of the cognitive variables and thought misinterpretations that have been extensively studied in Obsessive-Compulsive Disorder (OCD). However, further research is needed on the specific factors contributing to the development of TAF in patients with OCD. Since autistic traits and TAF are related to cognitive processes, we hypothesized in this study that autistic traits as well as schizotypal traits and obsessive-compulsive symptoms may be associated with TAF severity in OCD patients.

**Methods:**

In this cross-sectional study, eighty-three patients (aged 18 to 65) with OCD were assessed using the Yale Brown Obsessive-Compulsive Scale (Y-BOCS), Schizotypal Personality Questionnaire (SPQ), Autism Spectrum Quotient (AQ), Thought-Action Fusion Scale (TAFS), Beck Depression Inventory (BDI), and Beck Anxiety Inventory (BAI).

**Results:**

We found that attention switching, attention to detail, and communication dimensions of the AQ were associated with higher TAF-Likelihood/Self. There was a significant association between attention shifting and TAF-Moral, while attention to detail was significantly associated with TAF Likelihood/Others. Y-BOCS-Total (β = 0.338, *p* = 0.001), and cognitive-perceptual traits (β = 0.295, *p* = 0.018) were significantly associated with TAF-Moral. Likelihood/Self dimension of TAF was significantly associated with Y-BOCS-total (β = 0.386, *p* < 0.001), BDI (β = –0.333, *p* = 0.017), AQ-Total (β = 0.250, *p* < 0.001) and Cognitive-Perceptual schizotypal traits (β = 0.289, *p* = 0.016). The severity of TAF-Likelihood/Others was significantly associated with Y-BOCS-Total (β = 0.279, *p* = 0.012).

**Conclusions:**

We suggest that in addition to the severity of OCD and cognitive-perceptual traits, higher autistic traits may also contribute to increased levels of TAF-Likelihood/Self.

## Introduction

Obsessive-Compulsive Disorder (OCD) is characterized by the presence of obsessions and compulsions that cause significant anxiety, and distress. Obsessions often arouse anxiety in the individual, appear foreign to the sense of self, are repetitive in nature, and may sometimes be considered inappropriate. Compulsions are repetitive, ritualistic behaviors or mental acts that individuals feel compelled to perform in response to obsessions or to relieve distress [1]. Cognitive models of OCD propose that clinical obsessions result from catastrophic misinterpretations of meaningless but unwanted cognitive intrusions. The intrusion may be perceived as morally objectionable or likely to lead to an undesirable outcome. Thus, it is the interpretation, not the content, that causes increased anxiety and preoccupation [2].

Thought-action fusion (TAF) is one of the concepts related to cognition and thought misinterpretations that has been extensively studied in OCD. TAF is the belief that the probability of certain events will occur or be increased by certain thoughts (TAF-Likelihood). In TAF-Moral, unacceptable thoughts are morally equivalent to unacceptable actions [3]. Numerous studies have shown a relationship between TAF, especially TAF-Likelihood, and the severity of OCD [3, 4]. Cognitive processes such as TAF are thought to serve as an intermediary step between the emergence of obsessive thoughts and engagement in compulsive behaviors [2]. Rachman *et al*. [5] reported that TAF was significantly associated with obsession, guilt, even after depression was controlled for. Moreover, previously it has been found that experimentally induced TAF was associated with intrusive thoughts, discomfort, resistance, responsibility, and neutralization [6].

TAF-Likelihood appears to be associated with an extreme cognitive bias characterized by magical thoughts and schizotypal traits. A study examining the relationship between TAF and schizotypy observed positive associations between TAF-likelihood and magical thinking dimension of schizotypy. The positive association between schizotypal traits and TAF-Likelihood persists even after controlling for anxiety and depression. In contrast, TAF-Moral shows weaker associations with Obsessive-Compulsive Symptoms (OCS) and excessive magical thinking [7].

Schizotypy has been conceptualized as a three-dimensional construct consisting of positive, negative, and disorganized dimensions. The cognitive-perceptual factor encompasses unusual perceptual experiences, ideas of reference, paranoid ideation, and magical thinking. Interpersonal features include restricted affect, social anxiety, and lack of close friends. Disorganization factors include peculiar behaviors and speech [8]. Various studies have reported that the comorbidity rates between Schizotypal Personality Disorder (SPD) and OCD range from 5% to 50% [9, 10]. Additionally, Shan *et al*. [11] reported a co-occurrence rate of 3.33% between OCS and schizotypal traits in a student population. Emerging evidence suggests the existence of a distinct schizotypal subgroup within OCD. Earlier onset of the OCD, increased rates of learning disability, higher prevalence of comorbid disorders, and specific OCS such as aggressive and somatic obsessions and counting and arranging compulsions have been associated with higher schizotypy in OCD patients [9]. Positive symptoms of schizotypy, such as magical thinking, suspiciousness, distrust, and unusual beliefs, are more strongly associated with OCS [11].

Research is still needed on the specific factors contributing to the development of TAF in patients with OCD. One such factor may be autistic traits known to be associated with both schizotypy and OCD. Autism spectrum disorder (ASD) is characterized by restricted and repetitive patterns of behavior, interests, and activities, as well as differences in social communication and interaction. Autistic traits consist of five dimensions: social skills deficits, attention switching problems, attention to detail, communication problems, and lack of imagination. Autistic-like traits, also called the “broader autistic phenotype”, include reduced social skills, narrow interests, repetitive rituals, and increased attention to detail. These features are considered either to exist on the border between normal and pathological states, or to be milder manifestations of psychopathology [12]. Individuals with autism often exhibit disorganized cognitive profiles in nonverbal domains [13]. Previous studies indicated that individuals with higher autistic traits, but not meeting criteria for an ASD diagnosis are associated with reduced social cognition [14] and executive function [15], and enhanced local-focused perceptual processing [16].

Previous studies have shown a high comorbidity between ASD and OCD [17]. OCD and ASD exhibit similar clinical features, particularly in symptoms involving repetitive or stereotyped behaviors such as organizing, hoarding, tapping, or clicking, as well as rigidity and need for sameness [18, 19, 20]. In particular, the Autism Spectrum Quotient (AQ) subscales of attention shifting and communication emerged as significant predictors of OCD symptom severity, whereas attention to detail showed a low relationship with OCD severity [20].

To our knowledge, the relationship between autistic traits and TAF in OCD patients has not been investigated to date. Since autistic traits and TAF are related to cognitive processes, we hypothesized in this study that autistic traits as well as schizotypal traits and obsessive-compulsive symptoms (OCD) may be associated with TAF severity in OCD patients.

## Methods

### Participants

This study used a cross-sectional design conducted between January 2022 and January 2023. Although no a priori sample size calculation was performed, all the patients diagnosed with OCD who came to the outpatient and inpatient psychiatry clinics of the university hospital between January 2022 and January 2023 were consecutively evaluated, and those who fulfilled the inclusion criteria were included in the study. Therefore, 121 consecutive OCD patients were evaluated for participation. Approval from the ethics committee of the medical faculty was obtained (Registration: 2021/168). Informed written consent was signed by all participants included in the study. This study was conducted in accordance with the principles of the Declaration of Helsinki which ensures compliance with ethical standards in clinical research. All participants underwent an assessment using the Structured Clinical Interview for DSM-5 (SCID 5) [21]. The inclusion criteria were: having a OCD diagnosis according to DSM-5 criteria, being between 18 and 65 years of age, being of either gender, having the ability to understand study procedures, and having a Yale Brown Obsessive-Compulsive Scale (Y-BOCS) [22] score ≥16. Exclusion criteria for participants included schizophrenia, bipolar disorder, mental retardation, alcohol or other substances use disorders, tic disorder, and any neurological diseases. All OCD patients had not used medications for at least the last three months. Detailed information about the participants, such as age, gender, and level of education was collected using a sociodemographic form. Ten patients who had been taking medication and psychotherapy for the last three months, fifteen patients with a Y-BOCS score below 16, five patients diagnosed with bipolar disorder, and eight patients who did not agree to participate in the study were excluded from further assessment.

### Instruments

OCD severity was measured in 83 patients using the Turkish version [23] of the Y-BOCS [22]. Y-BOCS is one of the most widely used instruments for assessing the severity of obsessions and compulsions. This scale consists of 10 items rated from 0 (no symptoms) to 4 (extremely severe symptoms). Five questions are devoted to obsessions and five to compulsions. Total Y-BOCS scores range from 0 to 40 and rate severity based on the duration of symptoms, intrusion, associated anxiety, attempts at resistance, and the ability to successfully control obsessions and compulsions. Total scores consist of the sum of the scores obtained from the obsession (0–20) and compulsion (0–20) subscales. Cronbach’s alpha for the Turkish version of the Y-BOCS was 0.98, indicating good internal consistency.

For the assessment of the severity of schizotypal personality traits, the Turkish version of the [24] Schizotypal Personality Questionnaire [25] was used. The Schizotypal Personality Questionnaire (SPQ) is a 74 item instrument and the total score of the scale ranges from 0 to 74. Questions are answered with a yes or no. The SPQ has nine subscales screening DSM-III-R SPD diagnostic criteria, each containing 7–9 items. The scale consists of three factors: cognitive-perceptual, interpersonal, and disorganized schizotypy. The cognitive-perceptual schizotypy score is obtained by summing the scores for ideas of reference, bizarre belief-magical thinking, unusual perceptual experiences, and suspiciousness. The interpersonal schizotypy score is obtained by summing the scores for excessive social anxiety, lack of close friends, restricted affect, and suspiciousness. The disorganized schizotypy score is obtained by summing the scores for bizarre behavior and bizarre speech. Cronbach’s alpha for the Turkish version of SPQ total score was 0.91. Alpha values for the nine subscales ranged from 0.66 to 0.83.

The assessment of autistic traits was performed using the AQ [12]. The AQ is a 50-item self-administered questionnaire designed to measure autistic traits in adults and people as young as 16 years. Each item is scored from 1 to 4. Depending on item, responses 1 and 2 may be recorded as 1 point, or responses 3 and 4 may be recorded as 1 point. AQ total scores range from 0 to 50 points, with high scores indicating high autistic traits. The AQ has five subscales, each consisting of 10 items and representing a particular trait. Social Skill reflects confidence and comfort in social situations. Attention Switching refers to the capacity to shift focus between tasks or activities, and to adapt to changes in routine and unexpected events. Attention to Detail involves a heightened focus on details and patterns in the environment. Communication reflects the ability to engage in reciprocal communication, understand conversational cues, and interpret the nuances of social language. Imagination is related to imaginative thinking, including pretend play, hypothetical reasoning, and an appreciation for fiction or other imaginative scenarios. Participants indicate their level of agreement or disagreement on each item. The Turkish version of the AQ has been validated and found to be reliable among university students [26]. The Cronbach alpha value of the scale was 0.64, indicating acceptable internal consistency. Baron-Cohen and colleagues [12] found moderate-to-high Cronbach’s alpha values for the AQ subscale scores. Other studies have not found strong Cronbach’s alpha values. Gokcen *et al*. [26] suggested that this might explain the low Cronbach’s alpha values because Baron-Cohen and colleagues [12] created the AQ’s subscales without factor analysis. Furthermore, the authors reported that the Turkish version of the scale [26] was reliable because the AQ’s retest reliability coefficient of 0.72 was consistent the findings of Baron-Cohen and colleagues, who showed that Cronbach’s alpha coefficients were all moderate to high (Communication = 0.65; Social skill = 0.77; Imagination = 0.65; Attention to Detail = 0.63; Attention Switching = 0.67) [12].

The Thought-Action Fusion Scale-Revised (TAFS-R), developed by Shafran *et al*. [3], is a self-report measure that assesses the tendency to integrate thoughts into behaviors. The TAFS-R consisting of 19 items, uses a 5-point scale ranging from 0 (strongly disagree) to 4 (strongly agree). These items are subdivided into TAF-Morality, consisting of 12 items, and TAF-Likelihood, consisting of seven items. TAF-Likelihood is further subdivided into TAF- Likelihood/Others (four items) and TAF-Likelihood/Self (three items). Moral TAF refers to the belief that experiencing intrusive thoughts is inherently immoral or bad leaving the individual with a negative impression. Likelihood TAF, on the other hand reflects the belief that having unwanted thoughts increases the probability that the imagined event will actually occur. TAF-total scores range from 0 to 76 with higher scores indicating a stronger tendency toward TAF-like cognitions. Yorulmaz *et al*. [27] showed that the Turkish version of the TAF, which focuses only on the dimensions of morality and likelihood, is a reliable and valid instrument in nonclinical individuals. The internal consistency coefficient for the entire scale is 0.86, the Cronbach alpha coefficient is 0.75 for the first part (10 items) and 0.78 for the second part (9 items).

To measure the severity of characteristic emotional attitudes, intellectual, and physical symptoms of current depression, the Turkish version [28] of the self-report Beck Depression Inventory (BDI) [29] was used. The original BDI consisted of twenty-one questions about how the subject has been feeling in the last week. Each question had a set of at least four possible responses, ranging in intensity. A value of 0 to 3 is assigned for each answer and then the total score is calculated to determine the depression’s severity. Cronbach’s alpha for the Turkish version of the BDI was 0.80, indicating good internal consistency. To assess the severity of current anxiety symptoms, we used the Turkish version [30] of self-report Beck Anxiety Inventory (BAI) [31]. The BAI contains 21 questions, each answer being scored on a scale value of 0 (Not at all) to 3 (Severely—I could barely stand it). Higher total scores indicate more severe anxiety symptoms. The questions used in this scale are about common anxiety symptoms (such as numbness and tingling, sweating not caused by heat, and fear of the worst) that subjects have experienced in the past week (including the day you took the medication). Cronbach’s alpha for the Turkish version of the BAI was 0.93, indicating good internal consistency.

### Statistical Analysis

All statistical analyses were performed with Statistical Package for the Social Sciences (SPSS), version 22 (IBM Corporation, Armonk, New York, USA). The normality assumptions were tested using Kolmogorov-Smirnov Test and Skewness-Kurtosis values. Descriptive statistics were stated as mean (M) and standard deviations (SD) for continuous variables that were normally distributed. Additionally, median and interquartile range (P25, P75) values were reported for variables that were not normally distributed. Moreover, number (n) and percentage (%) values have been determined for categorical variables.

We used Spearman’s rank-order correlation analysis to explore the associations between clinical variables in our sample. Linear regression analyses were performed to determine factors that may be associated with TAF subscales in patients with obsessive-compulsive disorder. The variables significantly correlated with each TAF subscale were included as independent variables and subjected to linear regression analyses using the ‘enter’ method. Only variables that demonstrated significant bivariate associations with TAF were included in the regression models to avoid overfitting and to minimize multicollinearity. Beta coefficients and significance values were reported to assess the strength and significance of the associations. We also provided efficacy analyses that supported the sample size. We conducted post-hoc power analyses using G*Power (version 3.1.9.4; Franz Faul, Universität Kiel, Kiel, Germany) for each regression model (N = 83), with the observed R^2^ values, and the total number of predictors used in the regression model. In terms of multicollinearity, Variance Influence Factors (VIF) were calculated for each independent variable in our regression models. For each regression model, both *p*-values and 95% confidence intervals were reported for the regression coefficients.

## Results

### Descriptive Statistics and Spearman Correlations

Clinical and sociodemographic characteristics of OCD patients were presented in Table 1.

**Table 1.** **Sociodemographic and clinical characteristics of the sample**.

		OCD Patients (n = 83)
		N (%)
Gender		
	Female	58 (69.9)
	Male	25 (30.1)
Marital status		
	Single	46 (55.5)
	Married	35 (42.2)
	Widow/divorced/separated	2 (2.4)
Lifetime suicide attempt		14 (16.9)
Current Medication		65 (78.3)
		mean ± SD/median (P25, P75)
Age		30.62 ± 9.65
Education Level (years)		13.02 ± 3.77
Age at onset of OCD (years)		18 (14, 25)
Duration of OCD (years)		8 (4, 14)
BDI		21.57 ± 12.66
BAI		22.74 ± 14.27
Y-BOCS		
	Total	23.87 ± 5.43
	Obsession	12.60 ± 2.93
	Compulsion	11 (9, 13)
SPQ		
	Total	37.12 ± 18.87
	Cognitive-perceptual	13.30 ± 7.44
	Interpersonal	14 (9, 17)
	Disorganized	7 (3, 11)
AQ		
	Total	23.50 ± 5.87
	Social skill	4.98 ± 2.05
	Attention shifting	5.44 ± 1.98
	Attention to detail	5.32 ± 2.06
	Communication	3.77 ± 2.00
	Imagination	3.97 ± 1.84
TAF		
	Total	30.92 ± 17.92
	Moral	19.27 ± 11.36
	Likelihood-Self	4.71 ± 3.51
	Likelihood-Other	6.93 ± 4.52

OCD, Obsessive-Compulsive Disorder; Y-BOCS, Yale-Brown Obsessive-Compulsive Scale; BDI, Beck Depression Inventory; BAI, Beck Anxiety Inventory; SPQ, Schizotypal Personality Questionnaire; AQ, Autism Spectrum Quotient; TAF, Thought Action Fusion; SD, Standard Deviations. Note: Variables that are normally distributed were presented as mean and standard deviations. Variables that are non-normally distributed were presented as median and P25, P75 values.

The relationships TAF and other study variables in the study are reported at Table 2. Y-BOCS total scores were strongly related to all TAF dimensions, indicating that greater OCD severity is linked with stronger TAF. Additionally, TAF-Moral score was significantly correlated with SPQ total and all subscale scores and AQ-Attention Switching. TAF-Likelihood-Self Score was significantly correlated with SPQ total and subscale scores except SPQ-interpersonal. TAF-Likelihood-Self was also correlated with AQ total score and some subscale scores. TAF-Likelihood-Others was significantly correlated with AQ-Attention to Detail and SPQ total and subscale scores except SPQ-interpersonal.

**Table 2.** Correlation analyses between TAF and other clinical varibles (n = 83)*.

	TAF-M (R, *p*)	TAF-LS (R, *p*)	TAF-LO (R, *p*)	TAF-Total (R, *p*)
Age	–0.109, 0.327	–0.156, 0.158	0.051, 0.649	–0.072, 0.518
Age at onset of OCD	–0.009, 0.937	–0.191, 0.084	0.000, 0.997	–0.038, 0.733
BDI	**0.351, 0.001**	**0.222, 0.044**	0.206, 0.061	**0.326, 0.003**
BAI	**0.407, <0.001**	**0.371, 0.001**	**0.249, 0.023**	**0.409, <0.001**
Y-BOCS-Obsession	**0.442, <0.001**	**0.470, <0.001**	**0.377, <0.001**	**0.479, <0.001**
Compulsion	**0.411, <0.001**	**0.385, <0.001**	**0.284, 0.009**	**0.415, <0.001**
Total	**0.454, <0.001**	**0.456, <0.001**	**0.351, 0.001**	**0.475, <0.001**
SPQ-Interpersonal	**0.253, 0.021**	0.146, 0.189	0.118, 0.290	0.213, 0.053
Cognitive-Perceptual	**0.446, <0.001**	**0.404, <0.001**	**0.316, 0.004**	**0.455, <0.001**
Disorganized	**0.342, 0.002**	**0.313, 0.004**	**0.220, 0.045**	**0.339, 0.002**
Total	**0.392, <0.001**	**0.340, 0.002**	**0.264, 0.016**	**0.390, <0.001**
AQ-Social Skill	0.075, 0.499	0.180, 0.104	0.086, 0.440	0.107, 0.334
Attention Switching	**0.221, 0.045**	**0.299, 0.006**	0.130, 0.240	**0.218, 0.047**
Attention to Detail	0.201, 0.069	**0.294, 0.007**	**0.274, 0.012**	**0.240, 0.029**
Communication	0.117, 0.293	**0.229, 0.037**	0.056, 0.618	0.119, 0.285
Imagination	0.044, 0.696	–0.068, 0.539	–0.012, 0.914	–0.010, 0.926
Total	0.188, 0.089	**0.297, 0.006**	0.143, 0.198	0.193, 0.081

TAF, Thought Action Fusion; TAF-M, Thought Action Fusion-Moral; TAF-LS, Thought Action Fusion-Likelihood Self; TAF-LO, Thought Action Fusion-Likelihood Others; TAF-Total, Thought Action Fusion-Total; Y-BOCS, Yale-Brown Obsessive-Compulsive Scale; SPQ, Schizotypal Personality Questionnaire; AQ, Autism Spectrum Quotient. *Spearman’s Correlation Test. Bold indicates statistical significance (<0.05).

### Regression Analyses

Multiple regression analyses were conducted to evaluate the relationship between TAF subscales and other clinical variables (Table 3).

**Table 3.** **Results of linear regression analyses conducted to predict TAF (n = 83)**.

Dependent Variable	Independent Variables	B	SE	β	*p*	95% CI	VIF
TAF-M^a^	Constant	–7.510	5.526	-	0.178	–18.515, 3.494	-
	BDI	–0.100	0.120	–0.112	0.405	–0.339, 0.138	2.103
	BAI	0.141	0.104	0.177	0.180	–0.067, 0.348	2.025
	Y-BOCS-Total	0.707	0.209	0.338	**0.001**	0.290, 1.124	1.183
	AQ-Attention Shifting	0.527	0.575	0.092	0.362	–0.618, 1.671	1.197
	SPQ-Cognitive-Perceptual	0.451	0.187	0.295	**0.018**	0.078, 0.823	1.775
TAF-LS^b^	Constant	–5.743	1.890	-	0.003	–9.507, –1.979	-
	BDI	–0.092	0.038	–0.333	**0.017**	–0.168, –0.017	2.280
	BAI	0.050	0.032	0.204	0.118	–0.013, 0.114	2.036
	Y-BOCS-Total	0.250	0.063	0.386	< **0.001**	0.030, 0.269	1.165
	AQ-Total	0.149	0.060	0.250	**0.015**	0.124, 0.376	1.219
	SPQ-Cognitive-Perceptual	0.137	0.056	0.289	**0.016**	0.026, 0.248	1.695
TAF-LO^c^	Constant	–2.168	2.237	-	0.336	–6.622, 2.286	-
	BAI	0.014	0.039	0.044	0.726	–0.065, 0.092	1.540
	Y-BOCS-Total	0.232	0.090	0.279	**0.012**	0.053, 0.412	1.158
	SPQ-Cognitive-Perceptual	0.099	0.080	0.163	0.224	–0.061, 0.259	1.741
	AQ-Attention to Detail	0.362	0.241	0.166	0.136	–0.117, 0.841	1.199
TAF-Total^d^	Constant	16.830	9.019	-	0.066	–34.793, 1.134	-
	BDI	–0.216	0.200	–0.153	0.284	–0.614, 0.182	2.295
	BAI	0.291	0.169	0.232	0.089	–0.045, 0.628	2.080
	Y-BOCS-Total	1.233	0.335	0.374	< **0.001**	0.565, 1.902	1.187
	SPQ-Total	0.151	0.126	0.159	0.236	–0.100, 0.402	2.027
	AQ-Attention Shifting	0.816	0.957	0.091	0.396	–1.090, 2.723	1.297
	AQ-Attention to Detail	1.182	0.915	0.136	0.200	–0.641, 3.005	1.280

Adjusted R^2^ = 0.307^a^, 0.328^b^, 0.173^c^, 0.286^d^; Durbin-Watson = 2.265^a^, 2.298^b^, 1.842^c^, 2.170^d^. TAF, Thought Action Fusion; TAF-M, Thought Action Fusion-Moral; TAF-LS, Thought Action Fusion Likelihood Self; TAF-LO, Thought Action Fusion-Likelihood Others; TAF-Total, Thought Action Fusion-Total; Y-BOCS, Yale-Brown Obsessive-Compulsive Scale; SPQ, Schizotypal Personality Questionnaire; AQ, we; VIF, Variance Influence Factors; CI, confidence intervals. Bold indicates statistical significance.

Four separate regression models were tested, with TAF-Moral, TAF-Likelihood/Self, TAF-Likelihood/Others and TAF-Total serving as dependent variables in each model. Variables that showed association with each dependent variable at the bivariate level were included in the subsequent linear regression analyses. Given that Cognitive-Perceptual dimension of SPQ exhibited the highest correlation with the dependent variables, only this subscale was included in the regression analyses to mitigate concerns of multicollinearity. Similarly, in the second model, only AQ-Total was included to avoid multicollinearity. Additionally, it was decided to include only Y-BOCS-Total instead of its subscales in the analyses for the same reason.

Although we did not conduct an a priori power analysis, we did conduct post-hoc analyses in G*Power (F tests, linear multiple regression: fixed model, R^2^ deviation from zero; α = 0.05, N = 83). For all regression models, power was estimated to be high (1-β >0.95). In terms of effect size estimates (Cohen’s f^2^), Model a (TAF-Moral, R^2^ = 0.349) had a f^2^ of 0.54, Model b (TAF-Likelihood/Self, R^2^ = 0.369) had a f^2^ of 0.58, and Model c (TAF-Likelihood/Others, R^2^ = 0.214) had a f^2^ of 0.27 which can be interpreted as medium to large effect sizes. Also, a sensitivity analysis of the largest model (k = 5 predictors) demonstrated that the smallest detectable effect size in order to obtain 80% power was less than the observed effects, providing further assurance that we had an adequate sample size to detect the associations that we tested.

In the first model, TAF-Moral was included as dependent variable and the model explained the variance in the data well (Adj. R^2^ = 0.307, F = 8.261, *p* < 0.001). After controlling for other variables, Y-BOCS-Total (β = 0.338, *p* = 0.001) and cognitive-perceptual traits (β = 0.295, *p* = 0.018) were found to be significantly associated with TAF-Moral. In the second regression model (Adj. R^2^ = 0.328, F = 9.021, *p* < 0.001), the severity of TAF-Likelihood/Self was significantly associated with BDI (β = –0.333, *p* = 0.017), Y-BOCS-Total (β = 0.386, *p* < 0.001), AQ-Total (β = 0.250, *p* = 0.015) and cognitive-perceptual traits (β = 0.289, *p* = 0.016). In the third model (Adj. R^2^ = 0.173, F = 5.295, *p* = 0.001), only Y-BOCS-Total (β = 0.279, *p* = 0.012) was associated with TAF-LO. Furthermore, TAF-Total was significantly associated with only Y-BOCS-Total (β = 0.374, *p* < 0.001) when controlling for other variables. VIF values were calculated, all below 2.5 across the models, indicating no multicollinearity problem. Therefore, all predictors included in the final models can be considered independent enough to provide confidence in the estimation.

## Discussion

To our knowledge, these are the first results demonstrating an association between autistic traits and higher TAF levels in OCD patients. We found that attention switching, attention to detail, and communication dimensions of the AQ were associated with higher TAF-Likelihood/Self. There was a significant association between attention shifting and TAF-Moral, while attention to detail was significantly associated with TAF Likelihood/Others. In addition to the severity of OCD and cognitive-perceptual traits, total scores of AQ were significantly associated with the severity of TAF-Likelihood/Self.

Individuals diagnosed with ASD may exhibit impairments in neurocognitive mechanisms, such as reduced metacognitive efficiency, compared to typically developed children [32] and adults [33]. Metacognition has been identified as important for various aspects of cognition, including language acquisition, communication, social cognition, persuasion, focus, memory, problem-solving, self-control and self-directed learning [34]. Studies assessing metacognitive ability in autism have reported conflicting results, with some studies finding impaired metacognitive performance in autism [32, 33], while others finding no difference or better metacognitive performance in autism [35, 36]. In the metacognitive model [37], intrusions trigger metacognitive beliefs about the meaning and importance intrusions, specifically TAF. Our findings may therefore suggest that certain autistic traits that may be associated with metacognition, such as attention switching, attention to detail, and communication, have the potential to contribute to the development of high levels of TAF-Likelihood.

Additionally, alterations in social cognition, emotional regulation, motor control, and language development in autism relate to issues in the cingulate gyrus, amygdala, striatum, and cerebellum [38]. Neuroimaging studies show lower activation in the frontal, temporal, and parietal regions, as well as in frontostriatal and frontoparietal networks in autistic individuals [39]. Neuroscientific research on TAF indicates the involvement of the precuneus, lingual gyrus, caudate nucleus, and frontal-occipital areas during mentalization, empathy, and causal reasoning [40]. The default mode network (DMN), which often shows disruptions in autism, has reduced connectivity between the posterior and frontal areas [41]. More research is needed to clarify how neurobiological features shape the connection between autistic traits and TAF dimensions. Similarly, schizotypy links to brain activity in the middle temporal gyrus, temporoparietal junction and medial prefrontal gyrus, which are important areas of DMN [42]. Although studies have not confirmed shared brain abnormalities between TAF and schizotypy, future research may uncover their potential overlap.

The findings of this study partially confirm the relationship between TAF and schizotypal traits. Contrary to previous findings [7], all schizotypal traits, except interpersonal traits, show significant associations with both the likelihood and moral aspects of TAF. Although TAF-Moral and TAF-Likelihood initially appear to be separate constructs, some studies have elucidated how they may be related [3, 4]. For instance, individuals who believe that their thoughts increase the likelihood that a negative event will happen to someone else may make inferences about their own morality by taking into account the harm their thoughts may cause. Psychoanalytic explanations of OCD have suggested conceptual and clinically important links between magical thinking, pathological indecisiveness and doubt, and the tendency to engage in excessive moral reasoning [43]. TAF-Likelihood can be conceptualized as a specific type of magical thinking, while TAF-Moral may not be closely related to magical thinking because it involves an excessive personal interest in having a particular thought rather than an irrational inference of causality [7]. TAF-Moral resembles judgments regarding moral responsibility for thoughts or intentions, a construct that has been investigated in connection with religion. Preliminary studies including individuals from a variety of religious groups have identified positive correlations between religiosity and TAF, particularly TAF-Moral [44, 45]. TAF-Moral was more closely linked to higher levels of religiosity among Christians compared to Jews and Muslims [46]. Salkovskis *et al*. [47] have argued that religious institutions that explicitly impose moral standards on thought and behavior may contribute to the development of rigid and maladaptive beliefs about individuals’ thoughts. In other words, people who are highly religious perceive the presence and meaning of negative unwanted thoughts as more personally important, impressive, and immoral than non-religious or less religious individuals. Given that our sample consists of Muslim individuals, our findings may support the evidence for the relationship between schizotypal traits and TAF-Moral. As a result, we can suggest that both likelihood and moral TAF are associated with an extreme cognitive bias associated with schizotypal traits.

Our findings demonstrated that the moral and likelihood dimensions of TAF were significantly associated with the severity of both obsessions and compulsions. In addition, overall severity of OCD was significantly associated with higher moral and likelihood TAF. Although at the bivariate level, depressive symptoms were positively related to TAF-Likelihood/Self, this association became negative in the directionality in the multivariate model. This is potentially due to the suppressor effect that, while concurrently controlling for obsessive-compulsive severity, autistic traits, and cognitive-perceptual features of schizotypal nature, the shared variance between these interrelated factors obscures the direct contribution of depression to TAF-Likelihood/Self. Most previous studies have reported that TAF-Likelihood exhibited the most consistent associations with OCD symptoms [3, 4, 7]. However, findings regarding the TAF moral are inconsistent. Some studies have shown that TAF-Likelihood was more strongly associated with obsessionality than with the TAF-Moral, and that depression was more strongly related to TAF-Moral [48]. Some Turkish and Iranian studies reported that TAF-Moral scores showed a stronger correlation with OCS compared to TAF-Likelihood [46, 49]. They explained these differences by the different cultures and religious beliefs in their samples. TAF-Morality is evaluated especially in terms of religious obsessions [50]. Consistent with these findings, our study conducted in a Muslim sample, may indicate a potential relationship between OCD and TAF-Moral. However, it would be important to evaluate these findings in different and mixed ethnic and religious samples, especially to understand whether TAF-Moral is related to religion.

Several limitations of the current study should be noted. Although the achieved power values indicated adequacy of the sample size for the present models, we can suggest that the relatively modest sample size may still limit the ability to test statistical modeling assumptions and may reduce generalizability. Future research with larger or multi-center samples would help confirm the robustness of these findings. The cross-sectional design of our data precludes causal inferences about the relationships between autistic traits, schizotypal traits and TAF in patients with OCD. Therefore, prospective longitudinal studies are required to establish causal relationships between schizotypal and autistic traits and TAF in OCD patients.

Another limitation of this study is the use of self-report scales, which may be associated with recall bias, particularly in clinical populations. Participants may not respond accurately when responding to items, especially sensitive questions. To avoid retrospective reporting, future research could consider using clinician-administered assessments, observational, or longitudinal methods to validate findings and address these limitations. Additionally, although we did not make any formal corrections for multiple comparisons, we focused our analyses on predictors that are theoretically important. Furthermore, future research should also investigate TAF, schizotypy, and autistic traits in different populations beyond OCD, and also explore other potential mediators such as metacognition.

## Conclusions

Despite these limitations, this study aimed to provide evidence for research addressing the specific factors contributing to the development of TAF. Our findings may suggest that certain autistic traits related to metacognition, such as attention switching, attention to detail, and communication, could potentially contribute to the development of high levels of TAF-Likelihood. We may suggest that both likelihood and moral TAF are related to an extreme cognitive bias associated with schizotypal traits. Given that our sample consists of Muslim individuals, our findings may support evidence for the relationship between schizotypal traits and TAF-Moral. Our findings may encourage future research to investigate associations between autistic traits and TAF in OCD or other psychiatric conditions.

The present findings have important research and clinical implications. They offer new insights into the relationship between autistic traits as well as schizotypal traits and TAF in patients with OCD. Clinicians treating individuals with a predisposition to TAF should consider the role of schizotypal and autistic traits, which may be an important treatment target for both cognitive therapy and pharmacological treatment.

## Availability of Data and Materials

The data that support the findings of this study are available on request from the corresponding author.
